# Calcifediol Treatment Improves Skin Wound Healing in Vitamin D Deficiency Obese Rats

**DOI:** 10.3390/pharmaceutics18091175

**Published:** 2026-09-17

**Authors:** Bárbara Torrecillas-Baena, Victoria Pulido-Escribano, Marta Camacho-Cardenosa, María Dolores Carmona-Luque, Laura De los Santos Castillo-Peinado, Feliciano Priego-Capote, José Manuel Quesada-Gómez, Gabriel Dorado, María Ángeles Gálvez-Moreno, Antonio Casado-Díaz

**Affiliations:** 1 Unidad de Gestión Clínica de Endocrinología y Nutrición—GC17, Instituto Maimónides de Investigación Biomédica de Córdoba, Hospital Universitario Reina Sofía de Córdoba, 14004 Córdoba, Spain; b42tobab@uco.es (B.T.-B.); victoria.pulido@imibic.org (V.P.-E.); marta.camacho@imibic.org (M.C.-C.); md1qugoj@uco.es (J.M.Q.-G.); 2 Programa de Doctorado en Biomedicina, Universidad de Córdoba, 14071 Córdoba, Spain; 3 Dep. de Bioquímica y Biología Molecular, Facultad de Medicina y Enfermería, Universidad de Córdoba, 14004 Córdoba, Spain; b42calum@uco.es; 4 Dep. de Química Analítica, Campus Rabanales C3, Universidad de Córdoba, 14071 Córdoba, Spain; q22capel@uco.es (L.D.l.S.C.-P.); q72prcaf@uco.es (F.P.-C.); 5 Centro de Investigación Biomédica en Red de Fragilidad y Envejecimiento Saludable (CIBERFES), 14004 Córdoba, Spain; bb1dopeg@uco.es; 6 Dep. of Nursing, Pharmacology and Physiotherapy, University of Córdoba, 14004 Córdoba, Spain; 7 Dep. de Bioquímica y Biología Molecular, Campus Rabanales C6-1-E17, Campus de Excelencia Internacional Agroalimentario (ceiA3), Universidad de Córdoba, 14071 Córdoba, Spain

**Keywords:** calcifediol, obesity, wound healing, inflammation, vitamin D deficiency

## Abstract

**Background/Objectives:** Obesity is a major public health problem and a risk factor for numerous diseases. Furthermore, the high prevalence of 25-hydroxyvitamin D (25(OH)D) deficiency in obese individuals promotes a state of persistent inflammation and contributes to the development of hard-to-heal skin wounds. Although calcifediol is effective in correcting this deficiency, its effect on cutaneous wound healing in obesity has not been fully established. Thus, the objective of this work has been to evaluate the impact of calcifediol treatment on wound healing in an animal model of obesity with vitamin D deficiency. **Methods:** Wistar rats fed a high-fat, vitamin D-deficient diet were subjected to dorsal skin wounds. One group received calcifediol treatment for 14 days. Serum 25(OH)D levels, wound closure rate, histological parameters, and molecular markers related to inflammation were analyzed. **Results:** Calcifediol significantly increased serum 25(OH)D levels and accelerated wound closure compared to untreated animals. It also improved re-epithelialization, reduced the depth and extent of the lesions, and promoted collagen deposition. Furthermore, it increased the expression of anti-inflammatory markers (IL-10 and CD163) and decreased the infiltration of CD68+ macrophages, indicating a more efficient resolution of the inflammatory response. **Conclusions:** Calcifediol supplementation improves cutaneous wound healing in obesity with 25(OH)D deficiency by accelerating wound closure, promoting extracellular matrix remodeling, and modulating the inflammatory response, supporting its use as a complementary strategy to enhance tissue repair in conditions of obesity and vitamin D deficiency.

## 1. Introduction

The process of skin or cutaneous wound healing, and new tissue formation following injury, takes place through four precise and highly coordinated phases: hemostasis, inflammation, proliferation, and remodeling. For wound healing to be successful, all phases must occur in the proper sequence and at the right time. However, there are many factors that can interfere with one or more phases of the process, causing it to slow down. That may result in inadequate or deficient tissue repair and become a chronic hard-to-heal wound. Delayed acute wounds and chronic wounds may exhibit poor healing because they have not progressed through the normal phases of tissue regeneration and remain in a state of pathological inflammation [1,2].

Chronic ulcers place a huge burden on public health systems worldwide [3]. Throughout their lives, between 1% and 2% of the population, especially adults over 65 years old, will have suffered from chronic ulceration of mixed etiology [4]. Most of these chronic wounds are ulcers associated with underlying comorbidities such obesity, diabetes mellitus, nutritional deficiencies, and peripheral vascular disorders. The cost of the treatments required for this type of injury can exceed 2% of the national health care budget in regions such as Australia and Europe [5,6].

Obesity is an adiposity-based chronic disease that profoundly impacts both physical and mental health [7]. It is highly prevalent worldwide, with approximately 33% of the population suffering from overweight or obesity [8,9]. In addition, this disease affects and causes dysfunction, in virtually every organ and system of the body, leading to excess morbidity and mortality, with significant economic repercussions in developed countries [10].

Much attention has been paid to the consequences of obesity on the risk of other diseases such as type 2 diabetes mellitus, coronary heart disease, hypertension, dyslipidemia, respiratory problems, and certain forms of cancer. However, less attention has been paid to the effects of obesity on the skin [11]. Among the latter is the effect on wound healing. Indeed, obesity negatively affects wound healing, through a combination of chronic low-grade systemic inflammation and tissue hypoperfusion [12]. This is related to excess fatty tissue [13].

In this regard, delayed wound healing has been observed in patients with obesity, which may be attributed to changes that can easily disrupt the delicate balance on which the wound healing process depends. They include vascularization changes, reduced production of growth factors, and poor quality of granulation tissue [14]. As an example, the excessive tension at the wound edges in obese people favors dehiscence, which increases the risk of infections, as well as the formation of seromas or hematomas. All these factors facilitate and worsen the appearance of wound healing complications in obese people [15,16].

Another problem with excess fatty tissue in the body is that it can lead to chronic inflammation [17]. This can also affect the immune response and slow down the wound healing process. In obesity, macrophage differentiation during wound healing is impaired. This prolongs the inflammatory phase and, consequently, delays wound healing [18]. In addition, the accumulation of adipose tissue can cause poor circulation, limiting the blood supply to the wound. This can reduce the supply of nutrients and oxygen, which are essential for the healing process [15].

Interestingly, a wide range of chronic diseases and disorders such as obesity and increased severity of metabolic disorders such as insulin resistance, liver disease, hypertension, and hyperlipidemia have been associated with vitamin D deficiency [19]. Worldwide, approximately one milliard people suffer from this condition, and between 30% and 50% of the general population is vitamin D-deficient [20].

Vitamin D3 (cholecalciferol) is the threshold nutrient of the ‘vitamin’ D endocrine system (VDES) [21]. Although it can be obtained from some foods and supplements, the majority of vitamin D intake (80–90%) is obtained through its synthesis in the skin from 7-dehydrocholesterol after exposure to ultraviolet B (UV-B) radiation. Vitamin D is biologically inactive and requires two sequential hydroxylations, at positions 25 and 1α, to be activated. The first occurs in the liver, mainly by the microsomal cytochrome P450 family 2 subfamily R member 1 (CYP2R1) enzyme to form 25-hydroxyvitamin D (25(OH)D) or calcifediol. A second hydroxylation, by cytochrome P450 family 27 subfamily B member 1 (CYP27B1), occurs mainly in the proximal tubule of the kidney, but also in many cell types (skin, immune cells, lung, bone, placenta, etc.), to obtain the active metabolite, 1,25-dihydroxyvitamin D3 (1,25(OH)_2_D_3_) or calcitriol, which is the hormone of VDES [22]. Calcifediol, being a prohormone and cornerstone of VDES, is the metabolite with the highest blood concentration and longest half-life (two to three weeks) [23]. There is universal consensus that measuring the total blood concentration of 25(OH)D is the marker of the nutritional status of the vitamin D endocrine system [24].

25(OH)D deficiency is perhaps the most common nutritional deficiency in the world [25]. Organizations and societies around the world have published guidelines on recommended 25(OH)D serum levels and vitamin D intake [26]. However, the minimum thresholds for 25(OH)D in all age groups and populations remain a subject of debate, with no consensus on the definition of vitamin D sufficiency, insufficiency, or deficiency, as well as desirable serum levels of 25(OH)D [27]. Nevertheless, severe deficiency could be defined as 25(OH)D serum levels < 10 ng/mL, deficiency as 10 to 20 ng/mL, and levels between 20 and 50 ng/mL could be considered adequate and safe [26].

The main known actions of VDES are related to maintaining serum calcium, phosphorus homeostasis, and bone health [28]. Interestingly, numerous genetic, molecular, cellular, and animal studies strongly suggest that VDES signaling has many extra-skeletal effects [21]. These include the regulation of cell proliferation, immune and muscle function, reproduction and skin differentiation, and vascular and metabolic properties. Based on observational studies in human subjects, a deficient 25(OH)D status is associated with an increased risk of cardiovascular, autoimmune, and neuropsychiatric disorders, besides diabetes and cancer [19,29].

Additionally, VDES plays a fundamental role in the skin wound healing process. Thus, in addition to being a source of VDES metabolites, the skin is a target tissue for the physiological action of these metabolites [30]. Several studies have shown a correlation between vitamin D levels and the presence of hard-to-heal wounds [31]. In addition, 25(OH)D deficiency has been associated with slower skin healing, as well as an increased risk of developing chronic hard-to-heal wounds [32,33,34]. Thus, vitamin D deficiency has been associated with delayed re-epithelialization and extracellular matrix formation [35]. Indeed, VDES influences several key processes in wound healing. On the one hand, VDES affects the inflammatory and immune responses [36], which are crucial aspects in the first phase of the healing process [37]. It also participates in the proliferation and differentiation of fibroblasts and keratinocytes, comprising essential cells for skin regeneration [38,39,40]. Moreover, VDES is involved in calcium metabolism, which affects critical signaling pathways in the wound healing process [41,42]. Therefore, maintaining the function of the VDES mechanisms is essential for promoting rapid and effective healing of skin wounds, thereby preventing complications such as the development of infections or the wounds becoming chronic. Thus, it has been observed that in patients with chronic ulcers and vitamin D deficiency, treatment with cholecalciferol promotes healing [43].

Several studies have shown that treatment with the hormonal form of VDES, calcitriol (1,25(OH)_2_D_3_), promotes healing in animal models [44,45]. However, the effect of treatment with calcifediol (25(OH)D_3_) on skin wound healing, in models of obesity in conditions of vitamin D deficiency, has not been evaluated. Calcifediol, unlike calcitriol, can be commonly used in clinical practice to normalize 25(OH)D levels [46,47]. In this context, in the present study, we have selected calcifediol to evaluate how it may affect wound healing in an animal model of obesity with 25(OH)D deficiency.

## 2. Materials and Methods

### 2.1. Animal Model of Vitamin D Insufficiency and Obesity

The in vivo animal studies were conducted at the Experimental Animal Service (SAEX) of the University of Córdoba (Córdoba, Spain). The procedures followed the ARRIVE guidelines [48], being in accordance with the European Union Directive 2010/63/EU on animal experimentation. They were approved by the university’s bioethics and biosafety committee, and authorized by the Regional Government of Andalusia, Spain (authorization No. 16/10/2017/138).

In short, three-week-old male Wistar rats were selected to create a vitamin D-deficient obese rat model. For this purpose, the rats were fed a high-fat (70% fat, 16% protein, and 14% carbohydrates), vitamin D-deficient diet: C1090-70 Vit D3-deficient diet (high-fat) from Altromin (Lage, Germany) for 60 days. A standard diet was used as the control diet, namely C1000 Altromin with 13% fat, 20% protein, and 67% carbohydrates, including 500 IU/kg vitamin D3. Prior to wounding, the blood 25-hydrovitamin D metabolite was assayed to ascertain vitamin D deficiency.

25(OH)D serum levels were measured by a fully automated method, based on solid-phase extraction on-line, coupled to liquid chromatography with tandem mass spectrometry detection (SPE–LC–MS/MS) [49]. Sample preparation were carried out by SPE for cleanup/pre-concentration of 25(OH)D prior to determination in multiple reaction monitoring (MRM) mode. The intra-assay and the inter-assay coefficients of variation (CVs) for determination of 25(OH)D ranged from 4.6% to 8.1%.

### 2.2. Surgical Procedures and Treatments

The rats were divided into three groups of six animals each: (i) control, not obese, and not deficient in 25(OH)D (C); (ii) 25(OH)D-deficient obese (OD); and (iii) 25(OH)D-deficient obese treated with calcifediol (OD + 25(OH)D). For the surgical procedure, general anesthesia with isoflurane was performed. The anesthetized rats were placed in a prone position to remove hair from the dorsal side with an electric clipper. The skin was cleaned with physiological saline solution, and the rats were placed in a lateral position to make two wounds on the dorsum on each side of the midline, using a 10 mm disposable biopsy punch.

After surgery, 0.3 mg/mL of the analgesic Bupaq from Richter Pharma (Wels, Austria) was injected subcutaneously to relieve the animals of any post-surgical discomfort. The OD + 25(OH)D group was treated with calcifediol (hidroferol 0.1 mg/mL) from Faes Farma (Leioa, Spain) at a concentration of 5 µg/kg every 48 h for 14 days. The selection of the dose used was based on previous studies that have demonstrated its effectiveness in raising plasma levels of 25(OH)D to sufficient levels, as well as in producing effects related to VDES activation [50,51]. Calcifediol was diluted in olive oil, and 150 µL of the mixture was administered subcutaneously. Animals in the control and OD groups were treated with 150 µL of olive oil.

### 2.3. Measurement of Wound Size Changes

Postoperative photos were taken on days 0, 2, 6, and 10. The evolution of wound areas was analyzed using ImageJ software version 1.54 from the National Institutes of Health (NIH) (Bethesda, MD, USA). The following formula was used: Wound Closure (%) = [A_0_ − A_t_)/A_0_] 100, where A_0_ is the initial wound area, and A_t_ is the wound area at each time point.

### 2.4. Histological Studies

The animals were sacrificed 14 days after treatment with an overdose of anesthesia and exsanguination. With the aid of a disposable biopsy punch, the scar tissues were completely removed. For each animal, one wound was immediately frozen in liquid nitrogen for subsequent RNA and protein isolation, and the other wound was used for histological analyses. In the latter case, samples were dehydrated, embedded in paraffin, and cut into sections (~5 μm thick). Scar tissue depths, wound extents, epidermal thicknesses, wound inflammations, and vascularization were observed after hematoxylin and eosin (H&E) staining from Illinois Tool Works (ITW) Reagents (Glenview, IL, USA). To quantify the different parameters, photographs were taken under an optical microscope at 4×, 10×, and 40× magnification from at least two serial histological sections. Using the microphotographs taken at 4× and 10× magnification, scar tissue depth, wound extent, and epidermal thickness were quantified with the aid of the ImageJ, as indicated above. To quantify inflammation and vascularization in the scar tissue, at least four microphotographs were randomly selected from each histological section at 40× magnification. In each of these, the inflammatory cells and blood vessels present were counted using ImageJ software.

Masson’s trichrome staining was also performed to detect collagen depositions in the tissues at day 14. The Trichrome Stain kit from ScyTek Laboratories (West Logan, UT, USA) was used for this purpose. Collagen deposition in the wounds was quantified by analyzing images of the stained sections obtained under an optical microscope, using the Masson trichrome Color Deconvolution plugin in ImageJ.

### 2.5. Quantification of Gene Expressions by Quantitative Real-Time PCR

For gene expression analyses, skin samples were disaggregated in liquid nitrogen. RNA was isolated using the RNeasy Fibrous Tissue Kit from Qiagen (Germantown, MD, USA) following the manufacturer’s instructions. Nucleic acids were quantified with a DS11 spectrophotometer from DeNovix (Wilmington, DE, USA). Then, up to 900 ng of RNA was retrotranscribed into cDNA, using the iScript cDNA synthesis kit from Bio-Rad (Hercules, CA, USA), following the manufacturer’s instructions.

Quantitative real-time PCR (QRT-PCR) was performed on a CFX Connect instrument from Bio-Rad. Each PCR reaction was performed in a 10 µL volume containing 1 µL of cDNA, 10 pmol of each primer pair (Table 1), and 1× SensiFAST Sybr No-Rox Mix from Bioline (London, UK). The PCR amplification program included one cycle at 95 °C for 2 min (DNA denaturation) and 40–45 cycles of 95 °C for 5 s (DNA denaturation) and 65 °C for 30 s (primer hybridization and extension by DNA polymerase). The results were analyzed with CFX Maestro software version 2.3 (Bio-Rad). Glyceraldehyde phosphate dehydrogenase (*GADPH*) was used as the constitutive housekeeping gene.

### 2.6. Western Blots

For protein analyses, tissues were homogenized in liquid nitrogen and lysed with radioimmunoprecipitation assay (RIPA) buffer, supplemented with phenylmethylsulfonyl fluoride (PMSF) and protease inhibitors; both were obtained from Sigma-Aldrich, (St. Louis, MO, USA). Lysates were incubated in ice, centrifuged, and total protein concentration was determined using the detergent compatible (DC) Protein Assay (Bio-Rad). Equal amounts of protein (30 μg) were separated by sodium dodecyl sulfate-polyacrylamide gel electrophoresis (SDS-PAGE on 8–16% polyacrylamide gels from NuSeP (Germantown, MD, USA)) and transferred to polyvinylidene difluoride (PVDF) membranes. The latter were blocked with 5% skim milk in Tris-Tween 20 buffered saline (TTBS; 20 mM Tris-HCl pH 7.6, 150 mM NaCl, 0.05% Tween 20) and incubated overnight at 4 °C with primary antibodies anti-type I alpha 1 collagen (COL1A1) antibody [1:1000; ref. 72026 from Cell Signaling Technology (Boston, MA, USA)] and anti-type III alpha 1 collagen (COL3A1) antibody [1:1000; ref. AB6310 from Abcam (Cambridge, UK)].

After incubation with the corresponding secondary antibodies, proteins were detected using the avidin–biotin complex (ABC) and Clarity Western Enhanced Chemiluminescence (ECL) substrate and visualized with a ChemiDoc XRS+ system (Bio-Rad). Band acquisition and densitometric analyses were performed using Image Lab software version 6.0 from the same company. A stain-free technology was used for normalization, allowing total protein quantification in each sample and correction of the intensity of the target protein bands. Protein synthesis was expressed as relative levels, with the control group used as the reference.

### 2.7. Immunofluorescence

Deparaffinized tissue sections were placed in citrate buffer and boiled for microwave antigen retrieval. The sections were then washed with phosphate-buffered saline (PBS) for 5 min, treated with 0.3% PBS-Triton X-100 (10 min), and finally treated with 0.01% PBS-Triton X-100 (5 min). Samples were incubated with blocking buffer [bovine serum albumin (BSA), normal goat serum) (NGS) and horse serum] from Sigma-Aldrich at room temperature for one hour. Tissue samples were incubated with antibodies against CD68 [1:100, E3O7V from Cell Signaling Technology (Danvers, MA, USA)] at 4 °C overnight.

After washing with PBS, samples were incubated with Alexa Fluor 647-labeled goat anti-mouse IgG (H&L) [1:100, A21237 from Thermo-Fisher Scientific (Waltham, MA, USA)] in the dark at room temperature for 1 h. Samples were counterstained with 4′,6-diamidino-2-phenylindole (DAPI) for 10 min in the dark and mounted using CitiFluor AF4 antifade medium 17973-25 from Electron Microscopy Sciences (Morgantown, PA, USA). Fluorescence visualizations were performed using a Thunder Imager 3D Assay microscope by Leica Microsystems (Wetzlar, Germany). Finally, the areas corresponding to the scar tissues on each slide were analyzed, and the positive cells were quantified using Aivia software version 13 from Aivia Software (part of Leica Microsystems; Bellevue, WA, USA).

### 2.8. Statistical Analysis

Statistical analyses were performed using GraphPad Prism software version 9.0 from GraphPad Software (San Diego, CA, USA). Standard statistical methods were used for the calculations of the mean and standard deviations. The mean and standard error of the mean (SEM) were calculated from the experimental data. Analyses of variance (ANOVAs) and Fisher’s least-significant difference (LSD) multiple comparisons test were used to calculate *p*-values. Differences were considered statistically significant when *p* < 0.05.

## 3. Results

### 3.1. Serum 25(OH)D Values

Before inflicting wounds, the high-fat diet administered to the animals with induced obesity generated a statistically significant increase in body weight, compared to the control group, confirming the successful establishment of the experimental model of obesity. Likewise, the low-vitamin-D diet to which these animals were subjected caused a more than triple decrease in 25(OH)D serum levels, compared to control rats fed a standard diet: 4.8 ± 0.6 (*p* < 0.01) and 3.7 ± 0.8 (*p* < 0.01) vs. 15.0 ± 4.5 ng/mL. After 14 days of treatment with calcifediol, the rats showed a statistically significant increase in serum 25(OH)D levels, reaching values of 51.07 ± 1.66 ng/mL (Table 2).

### 3.2. Effect of Calcifediol Treatment on Wound Closure

**Figure 1:**
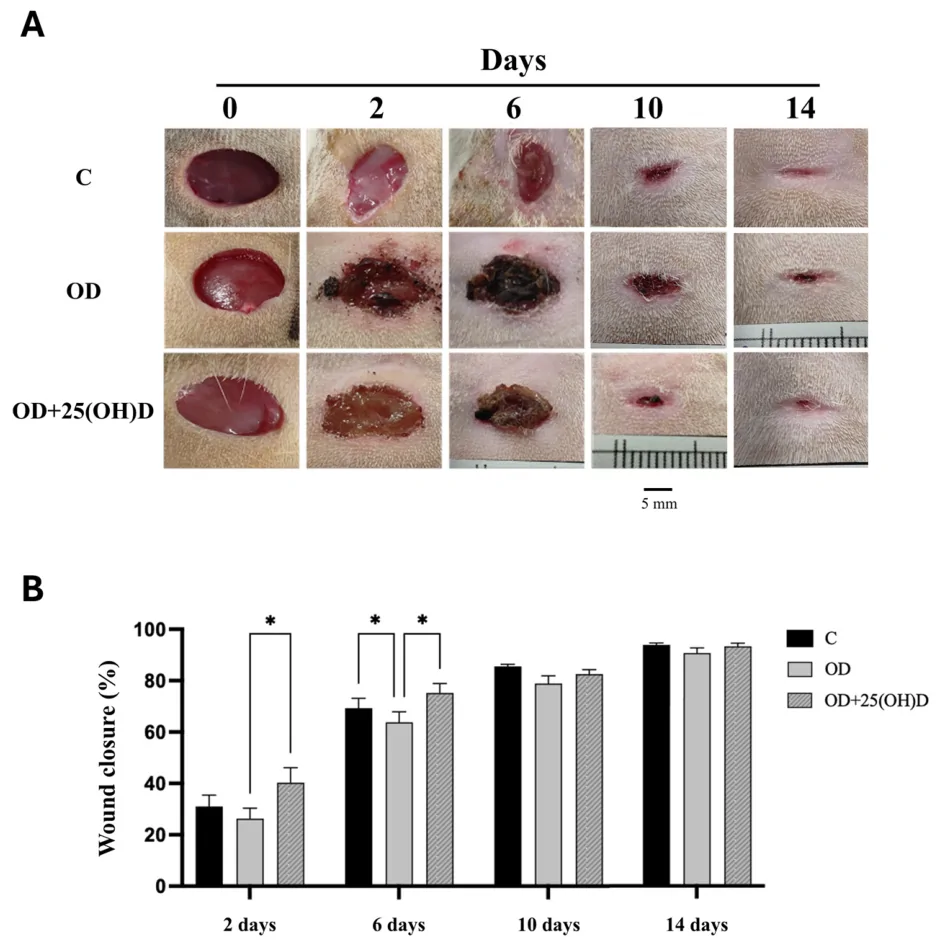
Temporal evaluations of wound closures. (**A**) Representative images of wounds at different times in experimental groups (see legend of Table 2). (**B**) Quantification of the percentage of wound closure in the different treatment groups at days 2, 6, 10, and 14 after the start of healing and treatments with 25(OH)D. * *p* < 0.05.

Figure 1 shows representative images of the evolution of wound closure in the different study groups, as well as their quantifications. After two days of treatment, obese animals deficient in 25(OH)D treated with calcifediol (OD + 25(OH)D) presented a greater degree of wound closure, with respect to the obese ones with 25(OH)D deficiency. On the sixth day, the control group (C) and the group of obese rats treated with calcifediol OD + 25(OH)D showed greater wound closure than the group of obese rats with 25(OH)D deficiency (OD). The treatments with calcifediol, in the obese animals deficient in 25(OH)D, produced the highest percentage of wound closures with 40.3% at day two, 75.2% at day six, and 82.6% at day 10.

### 3.3. Histological Analyses

**Figure 2:**
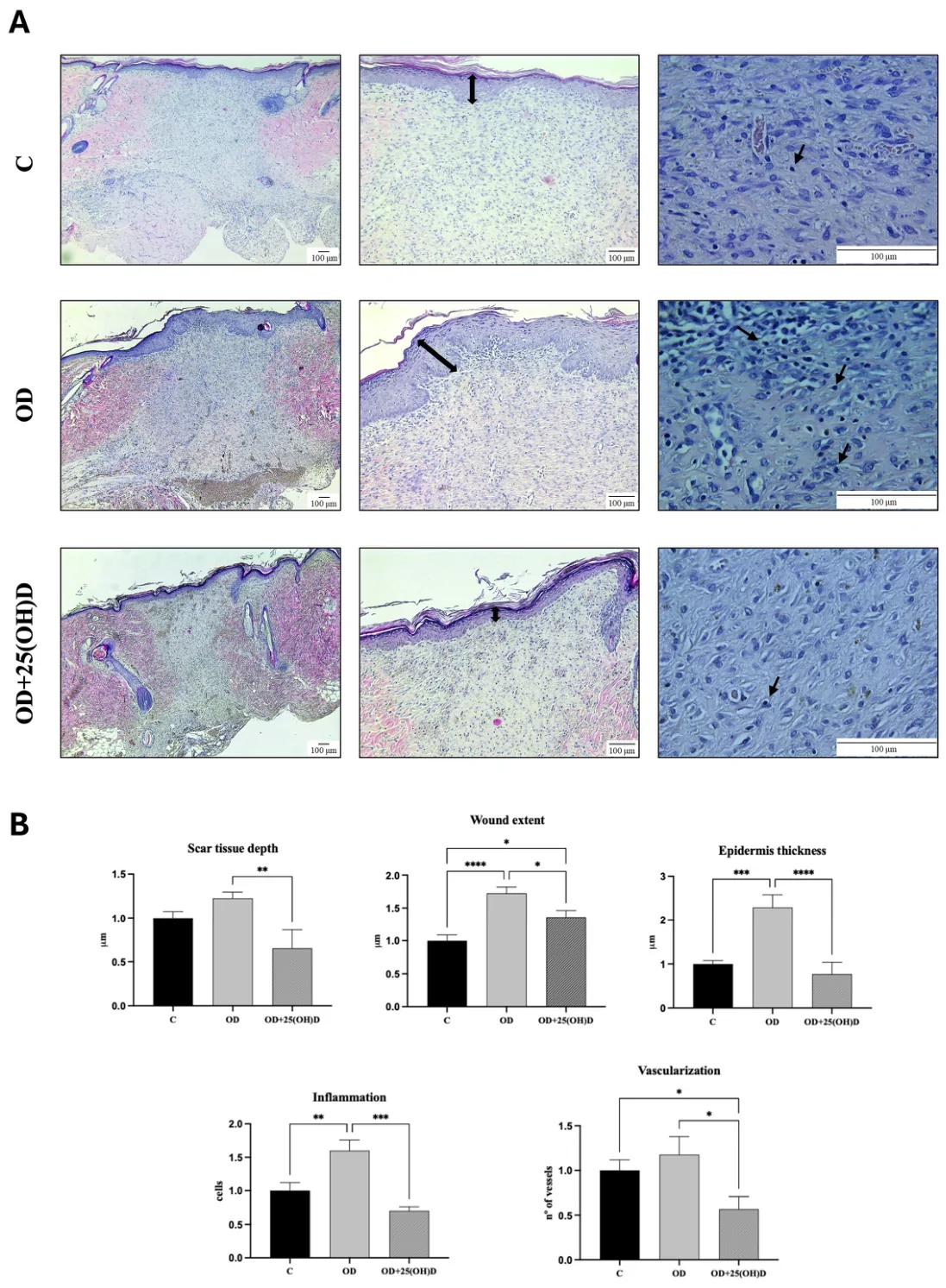
Representative images of histological sections of wounds stained with hematoxylin–eosin after 14 days of treatment. (**A**) Images from left to right correspond to each treatment taken at 4×, 10×, and 40×. The double arrows mark the thickness of the epidermis. The black arrows indicate areas with the presence of inflammatory cells. (**B**) Quantification of histological parameters in H&E stains of wound tissue samples obtained fourteen days after healing and treatment. * *p* < 0.05; ** *p* < 0.01; *** *p* < 0.001; **** *p* < 0.0001.

After fourteen days of treatment, wound tissue samples were histologically analyzed for various parameters using hematoxylin–eosin staining (Figure 2A). In this moment, wound closures were almost complete in all groups. Parameters related to epithelialization, inflammation, and vascularization were analyzed (Figure 2B). Regarding epithelialization, significant differences were observed. The wound extensions were greater in animals with obesity than in the control group, whilst treatments with calcifediol reduced the wound areas in those animals. In addition, the OD group showed a statistically significant increase in the wound depths of scar tissues, with respect to the calcifediol-treated group.

The epidermal thickness was also greater in OD, compared with control and OD + 25(OH)D rats. However, in the control and OD + 25(OH)D groups, a high degree of epithelialization was observed with a thinner epidermis and a lower depth and extension of the wounds. In the analyses of inflammation, obese animals with 25(OH)D deficiency showed an increase in inflammatory cells, which was not observed in the animals with obesity treated with calcifediol. Moreover, a significant decrease in vascularization could be observed in obese 25(OH)D-deficient animals treated with calcifediol, with respect to control and obese 25(OH)D-deficient ones (Figure 2B).

**Figure 3:**
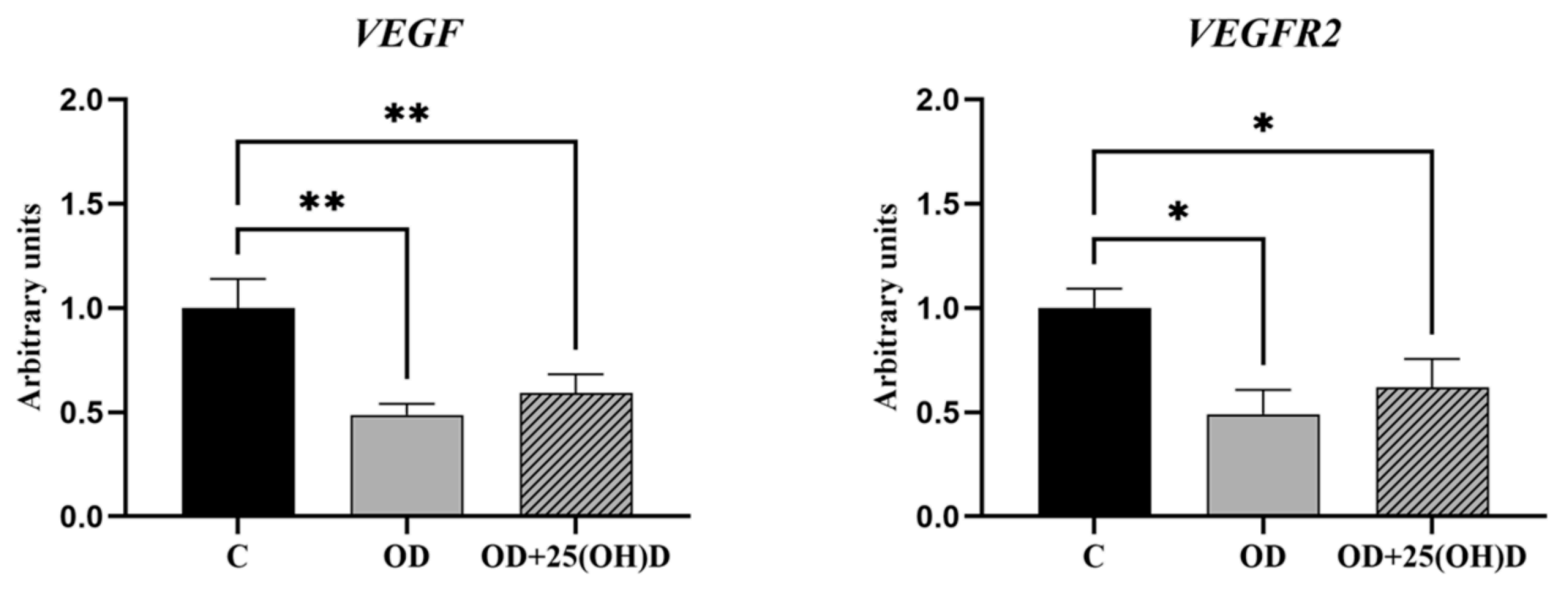
Gene expression of VEGF and VEGFR2 in scar tissues 14 days after treatment with calcifediol. Quantification of gene expression by QRT-PCR. * *p* < 0.05; ** *p* < 0.01.

With regard to the differences observed in vascularization, gene expression was studied in the scar tissue of vascular endothelial growth factor (VEGF), which encodes one of the main factors inducing angiogenesis, as well as its receptor, VEGFR2. For both genes, mRNA levels were found to be higher in the control group than in the obese group, regardless of whether the animals were treated or not with calcifediol (Figure 3).

**Figure 4:**
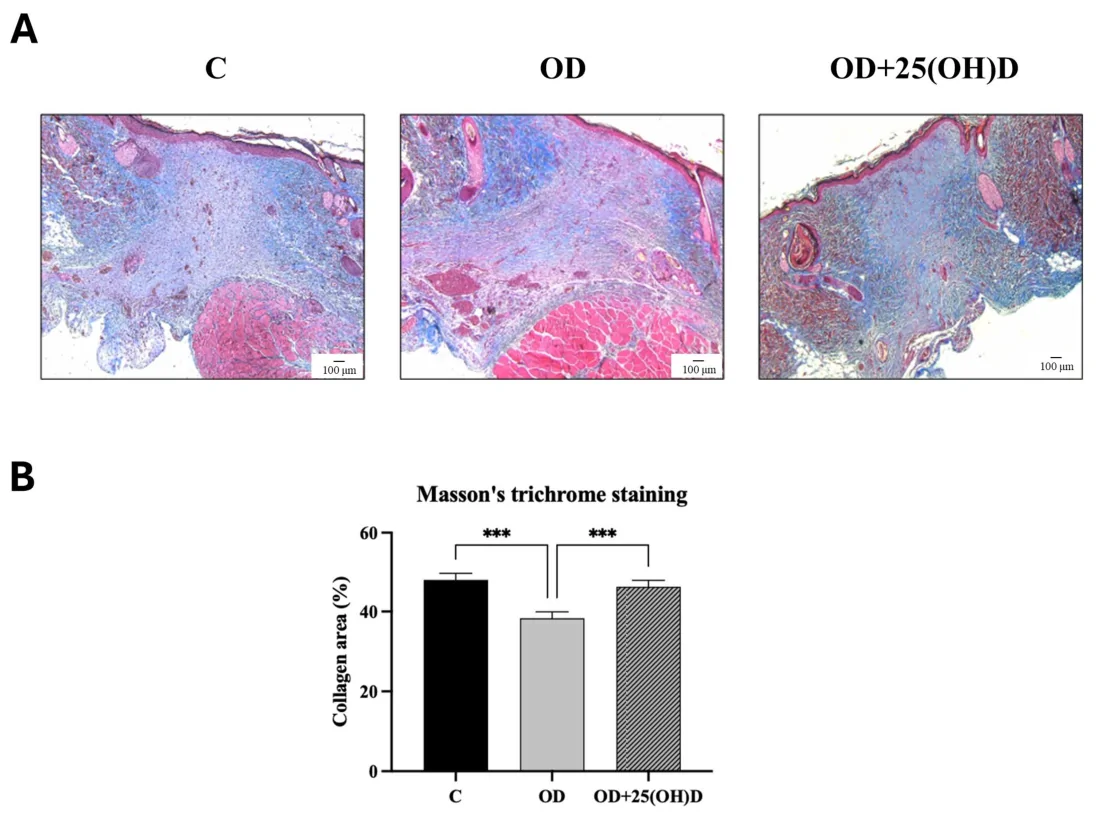
Masson’s trichrome staining. (**A**) Representative images of histological sections of wounds stained with Masson’s trichrome staining after 14 days of treatment. Collagen fibers are stained in blue. Images were taken at 4×. (**B**) Quantification of collagen deposition from Masson’s trichrome stain image analysis. *** *p* < 0.001.

Histological samples were stained with Masson’s trichrome to evaluate the collagen deposition in the extracellular matrix generated into the wound. The staining quantifications showed that the animals with obesity and 25(OH)D deficiency had less collagen in the scar tissue compared to the control group. However, treatment with calcifediol prevented this effect, with values like those of the controls (Figure 4).

**Figure 5:**
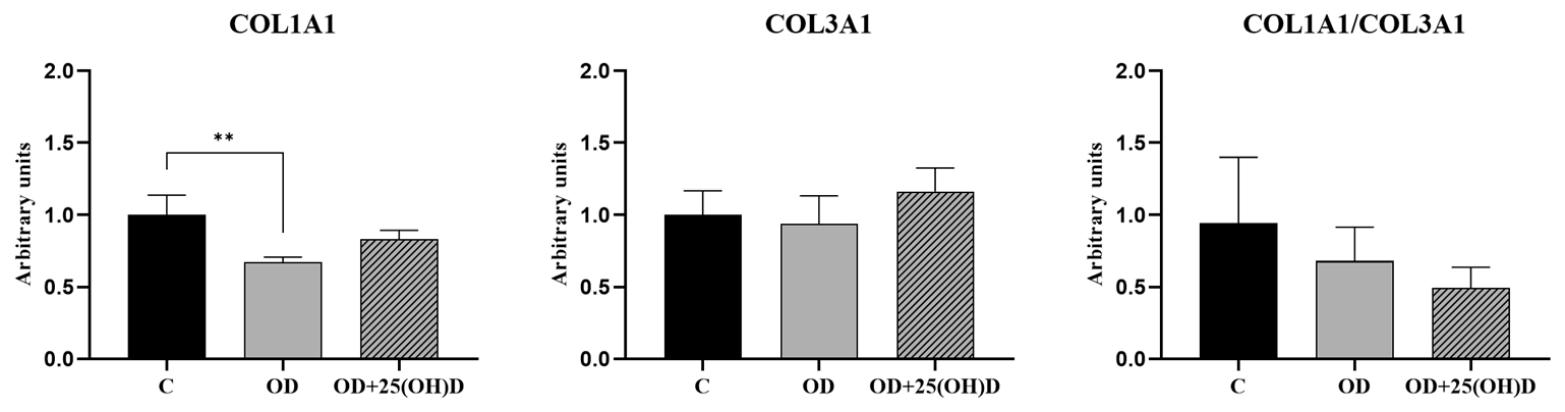
Protein synthesis of COL1A1 and COL3A1 in scar tissues 14 days after treatment with calcifediol. Quantification of COL1A1 and COL3A1 protein obtained from Western blot analyses (Figure S1) and COL1A1:COL3A1 ratio. ** *p* < 0.01.

The two main types of collagen generated during the formation and remodeling of the extracellular matrix are COL1A1 and COL3A1 [52]. Considering the results obtained, the protein synthesis of these two types of collagen was studied in the wounds. No statistically significant differences were observed between the different treatment groups in COL3A1. However, COL1A1 levels were higher in the control group than in the OD group, but not compared with the obese rats treated with calcifediol. The ratio of COL1A1 and COL3A1 synthesis provides information on the degree of maturity of the regenerated extracellular matrix. Analyses of this ratio showed that 14 days after the onset of wound healing, no significant differences were detected among the different groups of animals (Figure 5).

### 3.4. Calcifediol Increases the Expression of Anti-Inflammatory Markers in Obese Rats Deficient in 25(OH)D

**Figure 6:**
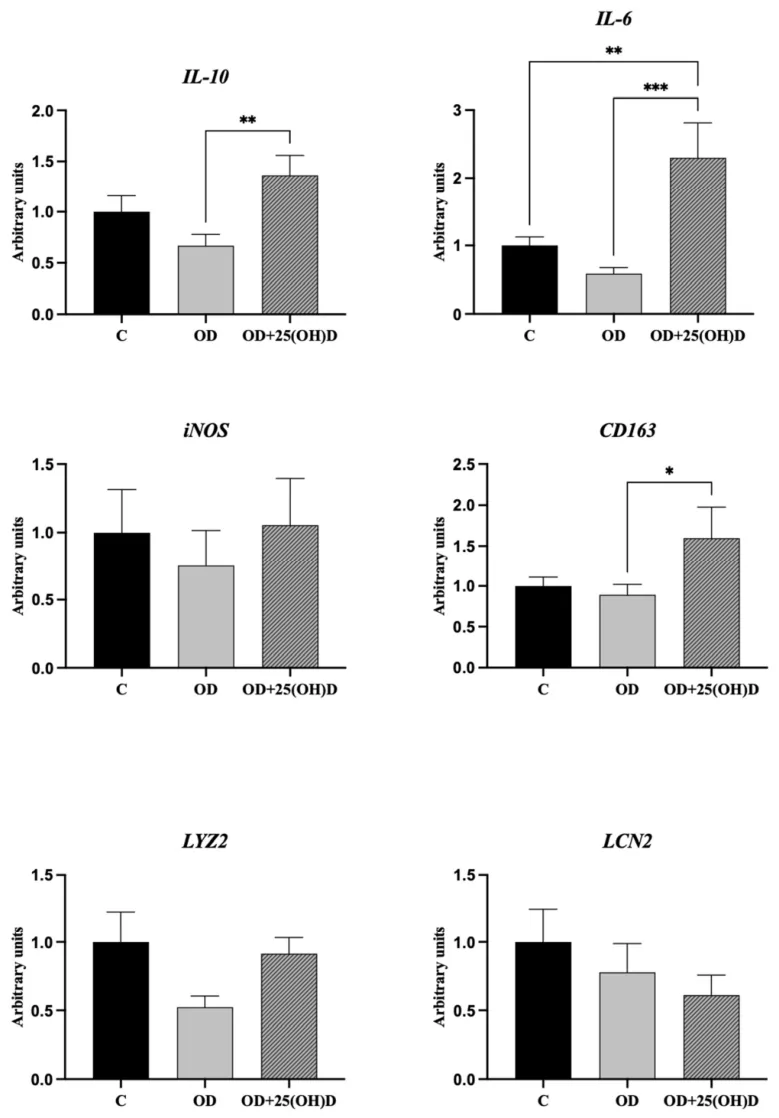
Gene expression of *IL-10*, *IL-6*, *iNOS*, *CD163*, *LYZ2*, and *LCN2* in scar tissues 14 days after treatment with calcifediol. Quantification of such gene expression by QRT-PCR. * *p* < 0.05; ** *p* < 0.01; *** *p* < 0.001.

The expression of different genes participating in the inflammatory process was studied at day 14 (Figure 6). In particular, the expression of interleukin-10 (IL-10), which encodes an anti-inflammatory cytokine, was statistically significantly increased in the calcifediol-treated groups, compared to untreated 25(OH)D-deficient animals. Similarly, in the group of obese animals with vitamin D deficiency that received calcifediol treatment, an increase in the expression of the IL-6 gene was detected. The expressions of the M1 (*iNOS*) and the M2 (CD163) macrophage marker genes were also evaluated. In the case of the former, no statistically significant differences were found between the groups. However, the expression of the latter was higher in obese animals with 25(OH)D deficiency treated with calcifediol. Finally, the expression of the lysozyme-2 (LYZ2) and lipocalin-2 (LCN2) genes was also studied. These genes may be expressed by other cell types, but they are considered markers of macrophages and neutrophils, respectively. Although slight differences were observed in the mean expression levels among the different experimental groups of animals, they were not statistically significant. Consequently, it cannot be concluded that obesity and vitamin D deficiency, with or without calcifediol treatment, alter the expression of these genes after 14 days of wound healing (Figure 6).

**Figure 7:**
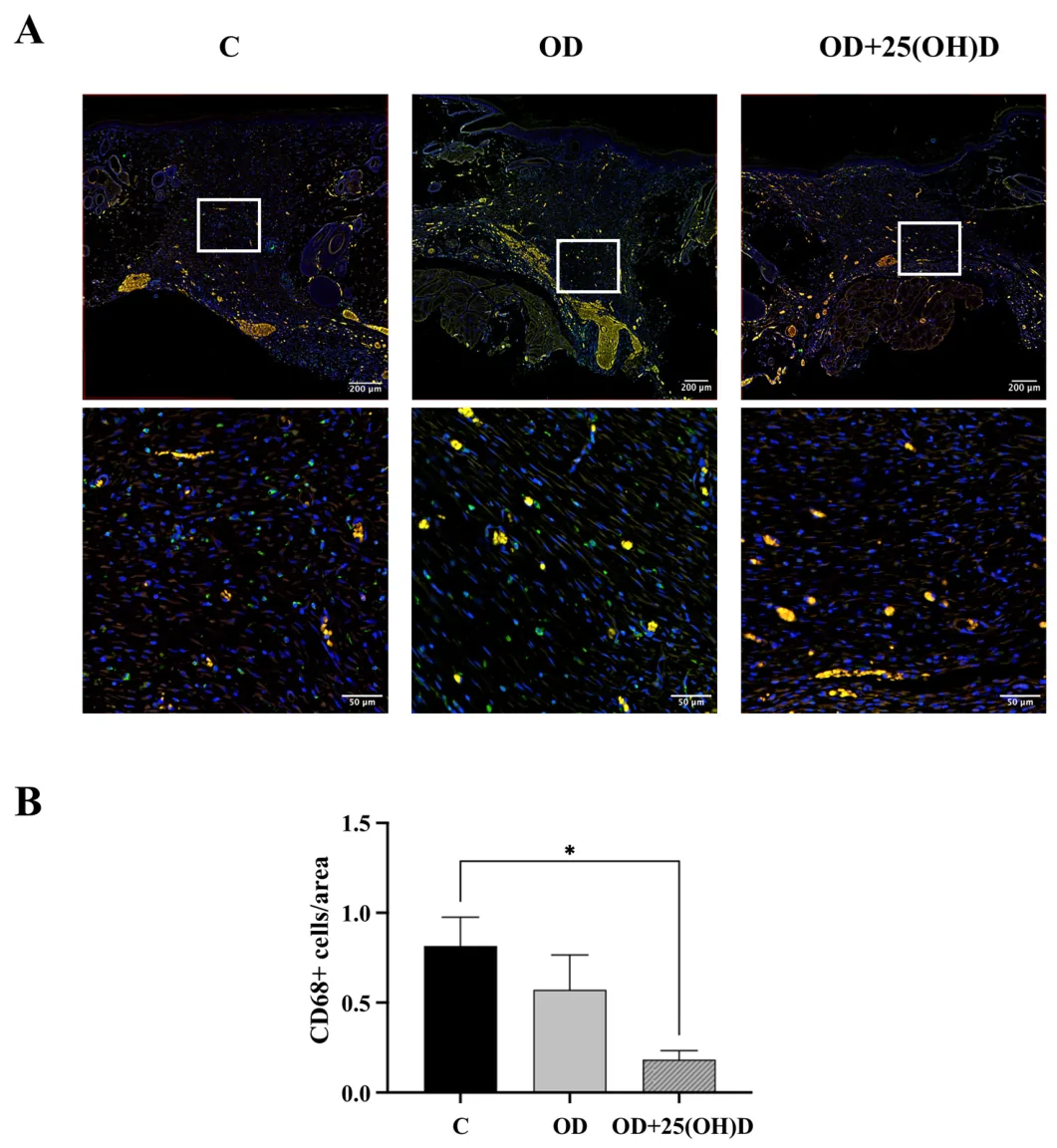
Immunofluorescence assays. (**A**) Representative images of CD68-labeled wound histological sections by immunofluorescence after 14 days of treatment. The lower images correspond to a magnification of the region surrounded by a white square in the upper images. (**B**) Quantitative analyses of the number of CD68+ cells marked by immunofluorescence on the wound. * *p* < 0.05.

Since studies on the expression of genes involved in inflammation showed treatment-dependent changes, as noted above, the macrophage marker CD68 was analyzed in the wound using immunofluorescence. This marker is specific to macrophages, but its overexpression has been associated with pro-inflammatory M1 macrophages [53]. The results showed that the group of animals treated with calcifediol had the lowest number of CD68+ cells in the scar tissue, indicating less infiltration of inflammatory cells into the wound bed (Figure 7).

## 4. Discussion

The development of hard-to-heal ulcers and the chronicity of wounds currently pose significant challenges for both clinicians and researchers in the field of regenerative medicine [54]. Obesity is one of the main risk factors for developing this type of wound [14]. The high prevalence of 25(OH)D deficiency in people with obesity is a very common condition in human clinical practice [9]. It is therefore necessary to assess possible links between these two conditions.

Since its discovery and isolation in the 1920s–1930s, vitamin D supplementation has been the most widely used strategy for improving the vitamin D status in the body [55], taking into account the risks of mutations and cancer involved in ultraviolet-light exposure. Cholecalciferol (vitamin D3) and, to a lesser extent, ergocalciferol (vitamin D2) are the most widely used compounds, and their effective, safe, and cost-effective use is well established. However, calcifediol represents a recent therapeutic alternative [56,57]. In this study, we used calcifediol as a treatment because it has pharmacokinetic advantages that may give it functional superiority over native vitamin D compounds for use in obesity [58]. Thus, the former is more hydrophilic and is absorbed following oral administration via the portal venous system. In addition, it does not require hydroxylation at position 25, a process that can be impaired during inflammatory processes, diabetes, and obesity [59].

In fact, calcifediol is available in high concentrations within a few hours of administration and remains stable [47], acting as a substrate for the synthesis of 1,25(OH)_2_D_3_ in the kidneys and other organs such as the skin, where it exerts its endocrine, paracrine, and autocrine effects [60]. Thus, the administration of calcifediol facilitates the biological actions of VDES before the system’s catabolic mechanisms [e.g., 24-hydroxylase, fibroblast growth factor 23 (FGF23), sulfatases, etc.] are substantially activated. These are maintained over time, serving the calcifediol as a substrate for the synthesis of 1α,25(OH)_2_D_3_. The latter is the VDES hormone that interacts with the intracellular vitamin D receptor (VDR), which is a transcription factor, a member of the nuclear receptor superfamily, which can regulate hundreds of genes on target cells [22].

The synthesis of 1,25(OH)_2_D_3_ from 25(OH)D occurs in the kidney, through a finely tuned hormonal regulation and via VDES metabolic pathways, contributing substantially to bone health and the body’s calcium and phosphorus homeostasis. However, other organs and tissues, such as the skin (which contains epidermal keratinocytes, fibroblasts, and dermal immune and vascular cells involved in wound repair), possess all the enzymatic machinery necessary for the autocrine and paracrine production of 1,25(OH)_2_D_3_. That is accomplished with 1α-hydroxylase (CYP27B1) and its catabolic inactivation via 24-hydroxylase (CYP24A1). That way, they regulate local levels of 1,25(OH)_2_D_3_, which acts on the VDR of skin cells involved in tissue repair [22,61]. However, vitamin D metabolism is altered in obese individuals [62,63]. Therefore, the response to vitamin D supplementation may also be affected.

In this study, we used a model of obese animals with 25(OH)D deficiency and skin wounds, mimicking a common scenario in human clinical practice, to assess how improving 25(OH)D status influences skin healing. Vitamin D (25(OH)D) deficiency in our model of obesity delayed wound healing, reduced extracellular matrix deposition, and altered the inflammatory response. Calcifediol supplementation accelerated wound closure, promoted collagen remodeling, and reduced inflammation. The 25(OH)D serum level in animals with obesity and 25(OH)D deficiency, following treatment with calcifediol during the 14-day healing period, increased significantly compared to untreated animals (51 ng/mL vs. 9 ng/mL; *p* < 0.001). Furthermore, it increased more than fourfold compared to animals fed a standard diet containing vitamin D (51 ng/mL vs. 13.6 ng/mL; *p* < 0.001). This indicates that the treatment with calcifediol at the administered dose enabled adequate levels of 25(OH)D to be achieved within a few hours [51,57].

In our model, the increase in 25(OH)D levels in animals treated with calcifediol was associated with a higher degree of wound closure compared to those with 25(OH)D deficiency. At the histological level, improved healing was confirmed in vitamin D-deficient animals treated with calcifediol. Thus, epidermal thickness and wound size decreased in treated animals after 14 days of healing, regarding untreated obese animals with 25(OH)D deficiency. This indicates that an increase in serum levels in 25(OH)D could improve epithelialization.

Following an injury, stem cells from the interfollicular epidermis and the hair follicle are activated to proliferate and migrate towards the wound. There, they acquire epidermal characteristics to re-epithelialize it and regenerate the epidermis [64]. VDR and the calcium-sensing receptor (CaSR) play a key role in these cells’ responses to the wound [65,66]. In fact, knocking out the *VDR* and/or *CaSR* genes in these cells delays wound healing [67]. In keratinocytes, the deletion of VDR, combined with reduced calcium availability or the deletion of CaSR, impairs the ability of the cells to undergo re-epithelialization [68]. 25(OH)D deficiency leads to the downregulation of *VDR* and *CaSR* gene expressions. On the other hand, appropriate levels of 25(OH)D improve intestinal calcium absorption and enhance the immune response [69]. Additionally, such physiological concentrations improve skin healing and upregulate *VDR* and *CaSR*, inducing wound healing [67].

On the other hand, it has also recently been demonstrated that the potent activating effect of 1,25(OH)_2_D_3_, on proliferation and differentiation of epidermal stem cells, is mediated by the activation of the phosphatidylinositol 3′-kinase (PI3K) signaling pathway [70]. Another pathway through which vitamin D may act to induce keratinocyte migration and re-epithelialization is the Hippo signaling pathway. Calcipotriol is a calcitriol analog which induces the expression of *TGF-β* gene by inhibiting this pathway, thereby promoting epithelial-to-mesenchymal transition (EMT) and keratinocyte migration [71]. The transcriptional activity of *VDR* activated by 1,25(OH)_2_D_3_ in keratinocytes is regulated by the presence of various coactivators and other transcription factors. These include beta-catenin, the mediator complex, and steroid receptor co-regulator complexes. The interaction of these factors with VDR affects the proliferation, migration, and differentiation of keratinocytes during re-epithelialization processes [67]. Taken together, these data support and help explain the results observed in our study regarding the positive effect of calcifediol treatment on wound epithelialization in obese animals.

It has been already demonstrated in Wistar rats that vitamin D3 promotes skin healing by increasing resistance and promoting epithelialization [72]. In fact, 1,25(OH)_2_D_3_ significantly increased the rate of early wound closure in ex vivo human skin explants [61]. Our data, therefore, confirm the essential role of maintaining adequate levels of 25(OH)D in tissue repair. In addition, after 14 days, a reduction in vascularization was also observed in the wounds of animals treated with calcifediol. This is also indicative of greater progress in the maturation of scar tissue in the skin remodeling phase [73]. However, when the gene expressions *VEGF* and its receptor *VEGFR2* were studied, it was observed that in obese animals, independent of serum 25(OH)D levels, there was a downregulation compared to the non-obese control group. Reduced *VEGF* expression during wound healing in obese animals has been described in various studies. It has been linked to impaired wound-healing capacity in such animals [74,75]. Furthermore, a decrease in the sensitivity of endothelial cells to VEGF has also been described in obesity models, as a result of a loss of VEGFR2 function [76].

Our results indicate that treatment with calcifediol did not change the effect of obesity on *VEGF* expression The data on it and its receptor expression did not correlate with the vascular density observed in the different treatment groups, as shown by histological analyses. This may be related to the fact that, in addition to endothelial cells, other cell types such as keratinocytes and macrophages also express VEGF receptors. Therefore, it is expected that, in addition to angiogenesis, VEGF plays a role in other processes during wound healing [77]. This still needs to be studied in greater depth to understand its potential implications for wound healing. That is particularly relevant in the context of obesity and diabetes as risk factors for chronic ulcers. It is also important to investigate its potential interaction with the effect of VDES during skin regeneration.

Other cells involved in skin wound healing that are affected by VDES are dermal fibroblasts. They express VDR and possess the enzymatic machinery required for the synthesis and catabolism of 1,25(OH)_2_D_3_ from 25(OH)D. They communicate and interact with adipocytes and vascular endothelial and inflammatory cells. In addition, the fibroblasts play an essential role in the synthesis of the extracellular matrix (ECM) and the regulation of keratinocyte migration [61]. A key feature of wound granulation tissue is the regeneration of ECM, which is essential for proper healing. Collagen deposition provides the necessary structural support for the formation of new tissue [78]. During the early stages of skin healing, the matrix is mainly composed of fibronectin 1 (FN1) and COL3A1, the latter being subsequently replaced by COL1A1, which is present from the seventh day post-injury [52]. Recent studies have shown that 25(OH)D deficiency negatively affects collagen deposition and extracellular matrix regeneration. Thus, lower gene expression of *COL1A1* and *COL3A1* in 25(OH)D-deficient animals, compared to controls, has been reported [35].

Similarly, in our study, Masson’s trichrome staining showed a decrease in collagen deposition in the wounds of obese 25(OH)D-deficient animals, which was reversed to values similar to the controls in the groups treated with calcifediol. Furthermore, in that group, there was a trend toward increased COL1A1 protein synthesis with respect to the obese 25(OH)D-deficient animals’ group. However, when the COL1A1:COL3A1 ratio was examined, no differences were observed between the two groups of obese animals and the control group. This could indicate that extracellular matrix maturation in the different groups was similar at that time. However, the fact that obese animals with vitamin D deficiency had lower collagen deposits suggests possible alterations in extracellular matrix synthesis, which could negatively affect wound healing.

The increase in collagen deposition following treatment with calcifediol may be related to the modulatory effect of vitamin D on fibroblast activity and extracellular matrix remodeling. Thus, under conditions of low TGF-β1 concentrations (2 ng/mL), it has been shown that vitamin D signaling, via the VDR, promotes collagen production and differentiation into myofibroblasts in dermal fibroblasts [79]. Likewise, more recent studies indicate that vitamin D3 exerted different effects on fibroblasts, depending on whether the cells are or are not in the process of migration. Thus, in cells induced to migrate, VDR activation by 1,25(OH)_2_D inhibited migration, the production of α-smooth muscle actin (αSMA), and the expression of type I collagen, thereby reducing excessive scar formation. However, in fibroblasts not induced to migrate, type I collagen expression significantly increased when they were treated with 1,25(OH)_2_D [61]. In our model, considering that, after 14 days of healing, the proliferation phase is well advanced, and the extracellular matrix in the wound is at the beginning of the remodeling process, it is possible to conclude that, at such a point, fibroblast migration is reduced and the increase in 1,25(OH)_2_D_3_, as a result of calcifediol metabolism, increases collagen production.

Therefore, optimizing vitamin D status may facilitate wound repair while minimizing scarring, which has implications for treating non-healing wounds and excessive scarring disorders. It is important to emphasize the importance of achieving optimal local levels of 25(OH)D that enable synthesis in keratinocytes and their progenitor cells, thereby helping to promote the restoration of the epidermal barrier and wound healing whilst minimizing the formation of scars and keloids.

Regarding the inflammatory process, in our model, 25(OH)D deficiency was associated with greater infiltration of inflammatory cells into wounds and lower expression of the anti-inflammatory cytokine IL-10. Both parameters were reversed in animals treated with calcifediol. This shows the importance of maintaining adequate 25(OH)D serum levels in obesity to improve the immune response and thus contribute to accelerated skin healing. Immune cells, including dendritic cells and macrophages as well as T and B cells, express the *VDR* gene. Interestingly, VDES has an anti-inflammatory effect [80] and plays a key role in regulating the immune response and inflammation, which are processes closely related to wound healing. During healing, macrophages play an essential role in the inflammatory phase and in the induction of tissue regeneration. At the beginning of healing, where inflammation predominates, they present a pro-inflammatory M1 phenotype. Subsequently, macrophages polarize into an M2 phenotype, associated with the resolution of inflammation and tissue repair [81].

It has been demonstrated, in a murine model, that supplementation with 1,25(OH)_2_D_3_ accelerated wound closure in diabetic animals, promoting the transition from M1 to M2 macrophages [45]. Furthermore, in elderly mice, supplementation with 1000 IU of vitamin D3 per week, for three months, improved skin healing, partly through a reduction in inflammation. In the wounds of treated animals, IL-10 levels increased and greater polarization of M1 to M2 macrophages was observed [44]. Consistent with these data, our results show that the obese animal group treated with calcifediol had reduced inflammation and showed a greater transition into the M2 phenotype. Thus, fewer CD68+ macrophages, associated with the M1 type, were detected in the group of obese animals treated with 25(OH)D. This translates into less inflammation in the scar tissue. In addition, treatment with 25(OH)D significantly increased the gene expression of anti-inflammatory markers, such as *IL-10* and *CD163*, characteristic of M2 macrophages. Therefore, the balance achieved with calcifediol treatment was to promote an anti-inflammatory and pro-regenerative state.

Regarding the expression of the gene encoding *IL-6*, it should be noted that this cytokine plays a dual role in inflammation. It can behave as a pro- or anti-inflammatory one, depending on the context [82]. In our study, increased expression of the *IL-6* gene was observed at day 14, at which the wound healing process is at an advanced stage. Therefore, at that time, *IL-6* could act as an anti-inflammatory marker. The reasoning for this is that its levels were significantly higher in the groups treated with calcifediol, especially in obese individuals. This coincided with the pattern of *IL-10* expression in these groups. In another study with mice, treatment with 1,25(OH)_2_D_3_ has been reported to decrease *IL-6* expression. However, in this case, expression was quantified on day five after the wounds were made. At that time, the inflammatory phase is still active and, contrary to what our results indicate, IL-6 might act as an inflammatory cytokine [44].

Our findings regarding the anti-inflammatory effect of calcifediol treatment are also supported by clinical studies. Thus, in human patients with diabetic foot, vitamin D deficiency has been associated with a higher concentration of inflammatory cytokines, mainly when there is an infection [83]. In addition, a recent meta-analysis based on seven randomized controlled clinical trials suggests that vitamin D supplementation promotes the healing of diabetic foot ulcers, partly through the reduction in inflammation [84].

Regarding the gene expression of *LYZ2* and *LCN2*, which are markers mainly associated with macrophages and neutrophils, respectively, no differences were observed between the different groups of animals. The expression of these genes has been positively correlated with blood glucose levels in diabetes models [74]. In our case, blood glucose levels in obese animals did not show significant differences from the control group. This may be related to the fact that, in our study, the high-fat diet was administered for eight weeks. This may be considered a short period of time to induce diabetes, in addition to obesity. In many studies using models similar to the one used in ours, it takes between 12 and 20 weeks on a high-fat diet to raise blood glucose levels [85]. This suggests that 25(OH)D serum levels, unlike glucose, do not affect the expression of such genes.

## 5. Conclusions

In conclusion, our findings confirm that 25(OH)D deficiency in animal models of obesity delays healing, reduces ECM deposition, and increases the inflammatory response. In contrast, calcifediol supplementation accelerates wound closure, promotes collagen remodeling, and modulates inflammation. Therefore, our results suggest that, to prevent and treat the development of chronic ulcers in patients with obesity, as well as providing specific care for each of the wound types, in accordance with the clinical guidelines used in best clinical practice, it is important to maintain adequate 25(OH)D serum levels. In cases of 25(OH)D deficiency, these levels can be effectively achieved by the administration of calcifediol [57,86]. However, the preclinical nature of our study is a limitation that must be taken into account when considering the translation of these findings into clinical practice. Therefore, future clinical studies are needed to evaluate the effect of calcifediol supplementation on individuals with obesity and vitamin D deficiency presenting chronic ulcers.

## Figures and Tables

**Table 1:** Primer sequences and product sizes.

Gene	Primer Sequence (5′ → 3′)	Amplicon (bp)
Interleukin-10 (*IL-10*)	GCGACGCTGTCATCGATTTCTC AGTGTCACGTAGGCTTCTATGC	153
Interleukin-6 (*IL-6*)	CTGCTCTGGTCTTCTGGAGTTC GCATTGGAAGTTGGGGTAGGA	177
Inducible nitric oxide synthase (*iNOS*)	ATTGGAGTCCGAGACTTCTGTG CCCGGTACTCATTCTGCATGTG	226
Cluster of differentiation 163 (*CD163*)	GAGGACGTCGGTGTGATCTG CCCCACTCTCCTTGGAATCTC	110
Lysozyme-2 (*LYZ2*)	ATGGGATGTCTGGCTACTATGG CACCAGTATCGGCTATTGATCTGA	151
Lipocalin-2 (*LCN2*)	CAGGCCCAGGACTCAACTCA CCAGGCCGACAACGAACCAC	115
Vascular endothelial growth factor A (*VEGFA*)	ATCTTCAAGCCGTCCTGTGTG ATGCTGCAGGAAGCTCATCTCT	162
Kinase insert domain receptor (*VEGFR2*)	TCCACCCAAGCTCAGCACACAA CCAACCACTCTGGGAACTGTG	195
Glyceraldehyde phosphate dehydrogenase (*GADPH*)	AGGTCGGTGTGAACGGATTTG AGACCATGTAGTTGAGGTCAATG	120

**Table 2:** Weight, glucose, and 25OH(D) values before and after surgery.

		C	OD	OD + 25(OH)D
Pre-surgery	Weight (g)	439.4 ± 13.03	536 ± 8.98 **	510.0 ± 12.92 **
	Glucose (mg/dL)	107.3 ± 9.15	131.4 ± 13.16	104.8 ± 8.94
	25OH(D) (ng/mL)	15.0 ± 4.5	4.8 ± 0.6 *	3.7 ± 0.8 *
Post-surgery	Weight (g)	419.8 ± 12.09	542.2 ± 9.74 **	490.2 ± 14.38 **
	Glucose (mg/dL)	105.4 ± 9.78	106.8 ± 15.10	107.6 ± 4.96
	25OH(D) (ng/mL)	13.58 ± 0.52	9.07 ± 0.5 *	51.07 ± 1.66 ** ^#^

Mean ± SEM are shown. C: control rats maintained on a standard diet. OD: obese rats maintained on a high-fat, vitamin D-deficient diet. OD + 25(OH)D: obese, vitamin D-deficient rats treated with calcifediol after the wounds were made. * *p* < 0.01; ** *p* < 0.001 vs. control. # *p* < 0.001 vs. OD.

## Data Availability

The original contributions presented in this study are included in the article. Further inquiries can be directed to the corresponding author.

## Supplementary Materials

The following supporting information can be downloaded at: https://www.mdpi.com/article/10.3390/pharmaceutics18091175/s1, Figure S1. Images of the Western blots used to analyze the expression of the COL1A1 and COL3A1 proteins in scar tissue from rats subjected to different treatments (C: control; OD: obesity and vitamin D deficiency; OD+25(OH)D: obesity and vitamin D deficiency, treated with 25(OH)D.
