# Supplementary material for: Calcifediol Treatment Improves Skin Wound Healing in Vitamin D Deficiency Obese Rats
Source: Pharmaceutics. 2026 Sep 17;18(9):1175. doi: 10.3390/pharmaceutics18091175 (PMC13610741; doi:10.3390/pharmaceutics18091175)
Supplement: Supplementary file 1 [file pharmaceutics-18-01175-s001.zip › pharmaceutics-4445618-supplementary.pdf]

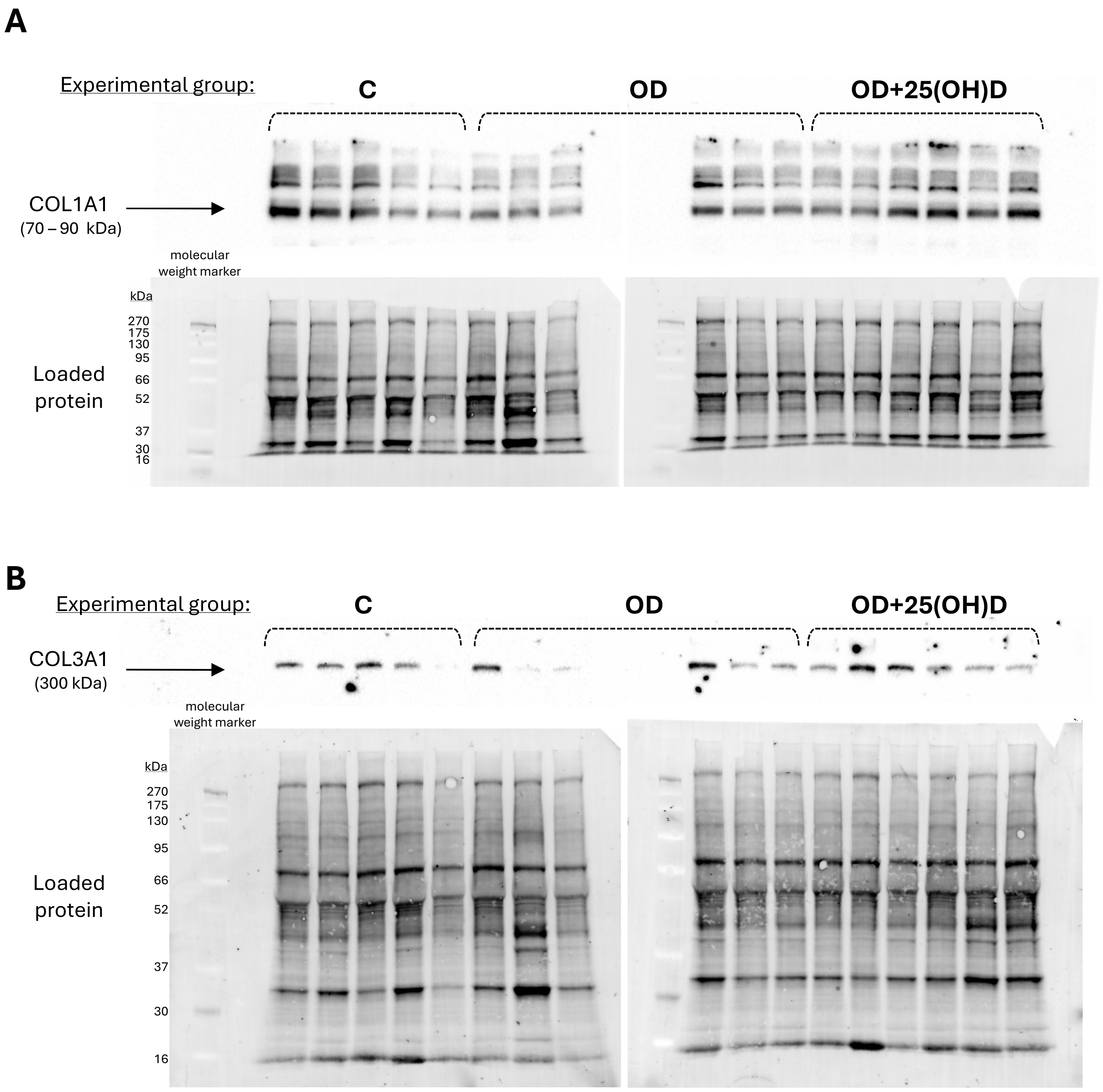

Figure S1. Images of the Western blots used to analyze the expression of the COL1A1 and COL3A1 proteins in scar tissue from rats subjected to different treatments (C: control; OD: obesity and vitamin D deficiency; OD+25(OH)D: obesity and vitamin D deficiency, treated with 25(OH)D. A, Western blot for COL1A1. The top image shows the protein bands obtained after hybridization with the anti-COL1A1 antibody (ref. 72026 from Cell Signaling Technology) and subsequent development. The lower image shows the membranes after applying stain-free technology to visualize the total protein loaded onto the gel. Based on this image, the total protein signal was quantified for each lane, and the resulting data were used to normalize the corresponding signal intensity value for the COL1A1 protein. B, Western blot for COL3A1. As in A, the top image shows the signal obtained after incubation with the anti-COL3A1 antibody (ref. AB6310 from Abcam) and subsequent development, and the bottom image shows the signal obtained for total protein using stain-free technology, which was used to normalize the COL3A1 values.
